# Metformin Improves Fertility in Obese Males by Alleviating Oxidative Stress-Induced Blood-Testis Barrier Damage

**DOI:** 10.1155/2019/9151067

**Published:** 2019-09-10

**Authors:** Jifeng Ye, Dandan Luo, Xiaolin Xu, Mingqi Sun, Xiaohui Su, Zhenhua Tian, Meijie Zhang, Chunxiao Yu, Qingbo Guan

**Affiliations:** ^1^Department of Endocrinology and Metabolism, Shandong Provincial Hospital affiliated to Shandong University, Jinan, Shandong 250021, China; ^2^Department of Endocrinology and Metabolism, Shandong Provincial Hospital affiliated to Shandong First Medical University, Jinan, Shandong 250021, China; ^3^Department of Endocrinology and Metabolism, Shandong Provincial Key Laboratory of Endocrinology and Lipid Metabolism, Jinan, Shandong 250021, China; ^4^Department of Endocrinology and Metabolism, The Second People's Hospital of Liaocheng, Shandong 252601, China; ^5^Department of Rheumatology and Immunology, Dongying People's Hospital, Dongying, Shandong 257000, China

## Abstract

**Background/Aims:**

Obesity, which is related to increased oxidative stress in various tissues, is a risk factor for male infertility. Metformin is reported to have an antioxidant effect; however, the precise role of metformin in obesity-induced male infertility remains unknown. The current study is aimed at exploring the effects of metformin and characterizing its underlying mechanism in the fertility of obese males.

**Methods:**

An obese male mouse model was generated by feeding mice with a high-fat diet; then, the mice were administered metformin in water for 8 weeks. Reproductive ability, metabolic parameters, and follicle-stimulating hormone (FSH) were assessed by cohabitation, enzymatic methods, and ELISA, respectively. Damage to the integrity of the blood-testis barrier (BTB), which ensures spermatogenesis, was assessed by transmission electron microscopy and immunofluorescence with a biotin tracer. Malondialdehyde (MDA), superoxide dismutase (SOD), and reactive oxygen species (ROS) were employed for the assessments of oxidative stress. BTB-related proteins were measured by immunoblotting. Nuclear factor *κ*B (NF-*κ*B) was assessed by immunofluorescence.

**Results:**

High-fat-diet-fed mice presented evident lipid metabolic disturbances, disrupted BTB integrity, and decreased reproductive function. Metformin alleviated the decrease in male fertility, decreased ectopic lipid deposition in the testis, and increased serum FSH levels. A further mechanistic analysis revealed that metformin ameliorated the high-fat-diet-induced injury to the BTB structure and permeability and restored the disordered BTB-related proteins, which might be associated with an improvement in oxidative stress and a recovery of NF-*κ*B activity in Sertoli cells (SCs).

**Conclusion:**

Metformin improves obese male fertility by alleviating oxidative stress-induced BTB damage. These findings provide new insights into the effect of metformin on various diseases and suggest future possibilities in the treatment of male infertility.

## 1. Introduction

Obesity is a complex metabolic disease that is determined by lifestyle factors, including environmental (food variety and intake, physical activity) and genetic factors. In recent decades, obesity has become a predominant health problem and has increased globally at an alarming rate [1]. Obesity increases the risk for hypertension, diabetes, cardiovascular disease, and some cancers [2–6]. Furthermore, the negative impact of obesity on the male genital system is gradually being recognized. According to clinical investigations, overweight men or men with obesity show a decline in sperm quality, an increase in sperm DNA damage, and a decrease in embryo implantation rates compared with men with normal body mass index (BMI) [7–10]. Additionally, animal experiments have shown that a high-fat diet can increase the male infertility rate and decrease sperm parameters [11–13]. Many previous studies involving mechanistic explorations have mainly focused on the effects of obesity or a high-fat diet on germ cells or Leydig cells. However, whether and how obesity damages Sertoli cells (SCs) or the blood-testis barrier (BTB) remains unclear.

SCs nurse developing germ cells and form the BTB between opposing SCs and adjacent Sertoli-germ cells [14–16]. The BTB is one of the tightest blood-tissue barriers, and it physically divides the seminiferous epithelium into the basal and apical compartments, in which different stages of germ cell development occur; these compartments are crucial to male fertility [17]. Several previous studies have demonstrated that various stimulators, such as high glucose [18], perfluorooctanesulfonate [19], cadmium [20, 21], amodiaquine [22], and bisphenol A [21], adversely affect male fertility via impairment of the BTB; however, whether obesity that is induced by a high-fat diet can provoke deleterious effects on the BTB has not been elucidated. The BTB comprises tight junctions (TJs), basal ectoplasmic specializations (basal ESs), gap junctions, and desmosomes. TJs, which coexist with basal ESs, are located between basal ESs and are reinforced by basal ESs [23]. The expression levels of TJ-related proteins (occludin and ZO-1) and basal ES-related proteins (N-cadherin and beta-catenin) are downregulated by exogenous stimulators, such as cadmium [20], perfluorooctanesulfonate [19], amodiaquine [22], and bisphenol A [24]. These studies indicate that TJs and basal ESs might be the molecular targets of exogenous stimulators.

Obesity can increase oxidative stress in the whole body [25], and an increase in testicular oxidative stress is a common feature in much of what underlies male infertility [26]. However, whether oxidative stress is involved in lipotoxicity-induced injury to SCs and the BTB is unclear. Metformin is a first-line hypoglycaemic agent. Beyond its glucose-lowering effects, metformin exhibits antioxidant properties in various tissues, an effect that is independent of its effect on insulin sensitivity, and acts to decrease lipid peroxidation [27, 28]. A recent clinical study has demonstrated for the first time that metformin improved semen quality in men with hyperinsulinaemia [29], but the underlying molecular mechanism is unclear. Moreover, a study in patients with polycystic ovarian syndrome (PCOS) has reported that metformin reduces angiogenesis through the nuclear factor *κ*B (NF-*κ*B) pathway [30]. However, whether metformin can improve the fertility of obese males and alleviate the damage of BTB in testis by inhibiting oxidative stress remains unknown.

In the present study, to determine the effect of metformin on fertility in obese males, an obese mouse model was induced with a high-fat diet, and metformin was administered. First, the effect of metformin on the reproductive ability of male mice was investigated. Alterations to the ultrastructure, BTB integrity, the expression of TJ proteins, and basal ES proteins were further assessed. In addition, the levels of oxidative stress and NF-*κ*B activity were measured to characterize the intracellular mechanism. This study demonstrates a crucial role of metformin in obesity-induced male infertility.

## 2. Materials and Methods

### 2.1. Animals and Treatment

Seven-week-old C57BL/6 male mice and nine-week-old C57BL/6 female mice were obtained from the Vital River Corporation (Beijing, China) and housed in a temperature- and humidity-controlled room (25 ± 2°C and 55 ± 10%, respectively) on a 12-hour light/dark cycle with free access to water and food. All experimental procedures were approved by the ethics committee of Shandong Provincial Hospital affiliated with Shandong University, and the methods were performed according to the approved guidelines.

As shown in [Supplementary-material supplementary-material-1], one week after feeding to adapt to the housing conditions, the male mice were randomly divided into two groups: the normal-diet group (N, *n* = 20) was fed a standard diet (Beijing Keao Xieli Feed Co. Ltd., China) in which 10% of the calories were from fat, and the high-fat-diet group (H, *n* = 30) was fed a high-fat diet (product #D12492, Research Diets Inc., New Brunswick, NJ, USA) in which 60% of the calories were from fat. Ten mice from each group were sacrificed at the end of the 8th week of feeding. The remaining mice in the N group were maintained on their standard diet (NN, *n* = 10), whereas the mice fed a high-fat diet (*n* = 20) were further subdivided into two subgroups. The first subgroup (HH, *n* = 10) was maintained on the high-fat diet, and the second subgroup (HH + MET, *n* = 10) was maintained on the high-fat diet with ~200 mg/kg body weight/day metformin (SFDA approval number H20023371, Sino-American Shanghai Squibb Pharmaceuticals Ltd., Shanghai, China) given in the drinking water [31]. The body weights of the mice were monitored at 5 pm every week during the entire feeding period (the adaptation week and the subsequent 8 or 16 weeks). All mice were sacrificed at the end of the 16th week of feeding.

### 2.2. Reproductive Ability Assay

To assess the reproductive ability of the male mice, at the 7th and 15th weeks after different feeding conditions, each male mouse was individually housed and mated with two female mice, which were randomly grouped by weight, for 5 consecutive days. On the second day, vaginal plugs were inspected to determine whether coitus had occurred. Female mice with vaginal plugs were moved into another cage and observed until the pups were born. The fertility of male mice was calculated according to the number of pregnant females. The number and weights of the pups were also statistically analyzed.

### 2.3. Blood Collection and Tissue Removal

At the 8th and 16th week, after being fasted for 8 h, all mice were anaesthetized by intraperitoneal injection with pentobarbital (40 mg/kg body weight) and sacrificed. Blood samples were obtained, and serum was extracted by centrifugation for the lipid profile and sex hormone analyses. For long-term storage, sera were kept at -80°C. Epididymal fat and testes were obtained immediately and weighed. The testis was fixed in modified Davidson's fluid (MDF) [32] for morphological analysis and immunofluorescence, fixed in 2.5% glutaraldehyde and 1% osmium tetroxide for ultrastructure analysis, or stored in liquid nitrogen for assessments by Oil red O staining, determination of mRNA and protein expression, and oxidative stress-related testing.

### 2.4. Serum Lipid Profile and Sex Hormone Analysis

The serum levels of total cholesterol (TC), triglycerides (TG), high-density lipoprotein-cholesterol (HDL-c), low-density lipoprotein-cholesterol (LDL-c), and glucose (Glu) were determined using enzymatic methods with an Olympus AU5400 automatic biochemical analyzer (Olympus Co. Ltd., Japan). The follicle-stimulating hormone (FSH) level in the serum was measured using an ELISA kit (Abnova, Taiwan, China) according to the manufacturer's protocol for each assay.

### 2.5. Oil Red O Staining

To determine testicular lipid accumulation, frozen sections of the testis (10 *μ*m) were fixed in 95% ethanol for 10 s, washed with distilled water for 10 s, stained with Oil red O (Changsha Guge Biotechnology Co. Ltd., China) for 10 min, washed again with distilled water for 10 s, and counterstained with haematoxylin for 30 s. Representative photomicrographs were captured using a microscope (Carl Zeiss, Germany).

### 2.6. Haematoxylin and Eosin (H&E) Staining Analysis

Testicular tissue samples were dehydrated in a graded series of ethanol solutions, embedded in paraffin, and coronally sectioned using a section cutter (Leica, USA) at a thickness of 4 *μ*m. H&E staining was performed for morphological observation using a system incorporated in the Carl Zeiss microscope.

### 2.7. Testicular Transmission Electron Microscopy (TEM) Analysis

To characterize the changes in testis at the ultrastructural level, the testis was dehydrated in a progressive ethanol and acetone solution, embedded in Epon 812, sectioned using a LKB ultramicrotome (LKB Instruments Inc., Sweden), stained with uranyl acetate followed by lead citrate, observed with a JEM-1200EX Transmission Electron Microscope (JEOL, Japan), and photographed.

### 2.8. BTB Integrity Assay

The integrity of the BTB assessment was evaluated using a biotin tracer as described previously [19]. In short, the mice were anaesthetized, and the testes were exposed before sacrifice. The gaps below the testicular tunica albuginea were injected with 50 *μ*l of EZ-Link Sulfo-NHS-LC-Biotin (10 mg/ml, freshly dissolved in physiological saline containing 1 mM CaCl_2_; Pierce Biotechnology Inc., IL, USA). After 30 min, the animals were euthanized, and their testes were frozen in liquid nitrogen in preparation for cryosectioning (10 *μ*m). The sections were fixed in 4% paraformaldehyde (PFA) for 20 min, washed with phosphate-buffered saline with 0.1% Tween20 (PBST) three times, blocked in 0.01 M phosphate buffer solution (PBS) containing 15% goat serum (Zhongshan Jinqiao Biotechnology Co. Ltd., China) and 1% bovine serum albumin (BSA, wt/vol) for 1 h, incubated with Alexa Fluor® 568-conjugated streptavidin (Life Technologies Corp.-Invitrogen, CA, USA) and 4′-6-diamidino-2-phenylindole (DAPI, blue) (Invitrogen, UK) for 2 h at room temperature, and washed again with PBST five times (5 min per time). After mounting with antifade mounting medium P0128 (Beyotime Biotechnology, China), the sections were analyzed by fluorescence microscopy (Axio Imager A2, Carl Zeiss, Germany).

### 2.9. Immunoblotting Analysis

Frozen testes were lysed in RIPA buffer with a protease inhibitor cocktail, PMSF and sodium orthovanadate (Santa Cruz Biotechnology Inc., CA, USA), to extract the whole protein. The cytosol and nuclear protein were obtained using the Nuclear and Cytoplasmic Protein Extraction Kit (CWBIO, China) according to the manufacturer's instructions. The protein concentration was quantified using a BCA protein assay kit (Pierce Biotechnology Inc., IL, USA). A total of 120 *μ*g whole protein or 60 *μ*g nucleoprotein lysates was resolved by SDS-PAGE using 8% denaturing polyacrylamide gel, electrotransferred to a PVDF membrane (Millipore, America), and blotted with specific antibodies, namely, anti-ZO-1, anti-occludin, anti-N-cadherin, anti-beta-catenin, anti-nectin2, and anti-NF-*κ*B/p65, with anti-beta-actin or anti-LaminB1 as a loading control for cytosolic proteins or nuclear proteins, respectively. The appropriate secondary antibodies conjugated to horseradish peroxidase (HRP) (Amersham, Little Chalfont Bucks, UK) were used at a 1 : 5000 dilution. The membranes were visualized using the HyGLO HRP detection kit (Denville, NJ, USA). The antibodies are shown in Table 1.

### 2.10. Isolation of RNA and Real-Time Polymerase Chain Reaction (PCR) Analysis

Total RNA was isolated from the tissues using an RNeasy Total RNA Isolation Kit (TaKaRa Bio Inc., Japan) and reverse-transcribed into cDNA (TaKaRa Bio Inc., Japan). Then, SYBR Green (DBI, Germany) quantitative PCR analysis reactions were performed using the Roche LightCycler 480 Detection System (Roche, Belgium). Each reaction comprised 10 *μ*l of SYBR Green, 1 *μ*l of cDNA, 1 *μ*l of each primer pair (10 *μ*mol/*μ*l), and 8 *μ*l of distilled water. The beta-actin gene was simultaneously detected as an endogenous reference. The relative gene expression levels were quantified using the 2^−ΔΔt^ method, and the results are expressed as the fold change relative to the endogenous reference. The primer sequences are listed in Table 2.

### 2.11. Malondialdehyde (MDA), Superoxide Dismutase (SOD), and Reactive Oxygen Species (ROS) Measurements

Assay kits for MDA and SOD were provided by Beyotime Biotechnology (China), and an assay kit for ROS was provided by Nanjing Jiancheng Bioengineering Institute (China). The MDA content, SOD activity, and ROS levels were measured using the kits according to the manufacturer's instructions, and these measurements were normalized to total protein.

### 2.12. Immunofluorescence

The testicular tissues were fixed with MDF and embedded in paraffin blocks. The sections were deparaffinized, and antigen retrieval was performed with Tris-EDTA (pH = 9.0). After blocking with 10% normal goat or donkey serum (Zhongshan Jinqiao Biotechnology Co. Ltd., China) for 60 min at room temperature, the slides were incubated overnight at 4°C with ZO-1 (1 : 25) or NF-*κ*B/p65 (1 : 250) primary antibody. For the negative control, 0.01 mol/l PBS was added instead of the primary antibody. The tissues were then incubated with the Alexa Fluor® 488F (ab′) 2 fragment of goat anti-rabbit IgG (H+L) (Thermo Fisher Scientific, USA) or with a donkey anti-Rabbit IgG (H+L) highly cross-absorbed secondary antibody, namely, Alexa Fluor® 555 (Thermo Fisher Scientific, USA) at a 1 : 1000 dilution at room temperature for 1 h. The nuclei were stained with DAPI, and the coverslips were sealed to the microscope slides using the mounting medium P0128. The specimens were imaged under a Carl Zeiss fluorescence microscope.

### 2.13. Statistical Analyses

Significant differences between the obtained values (mean ± SD) were determined by an independent sample *t*-test or one-way analysis of variance (ANOVA) followed by the least significant difference (LSD) multiple comparison test. A *P* value of <0.05 was considered significant.

## 3. Results

### 3.1. High-Fat Diet Increases Body Weight, Induces Abnormalities in Glucose and Lipid Metabolism, and Downregulates Fertility in Male Mice

**Figure 1:**
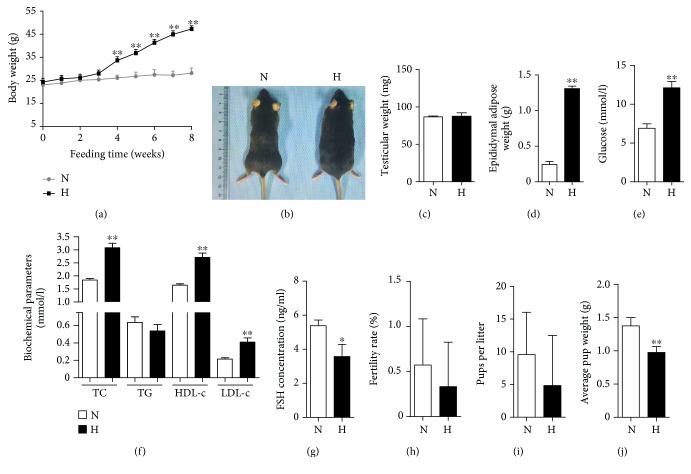
High-fat diet induces glycolipid metabolic abnormalities and damages the reproductive function of male mice. (a) Comparison of the time-dependent changes in body weight between the normal-diet-fed (N) and high-fat-diet-fed (H) groups during the 8 weeks of feeding. (b) Representative picture of normal-diet and high-fat-diet mice on the 8th week. Comparison of testicular weights (c) and epididymal adipose tissue (d) in the N and H groups on the 8th week. The serum glucose (e), biochemical parameters (f), and FSH levels (g) were assayed on the 8th week. *n* = 10 for each group. Comparison of fertility, including the fertility rate (h), number of pups per litter (i), and average pup weight (j) on the 8th week of feeding in the N and H groups. For the fertility assay, *n* = 7 males were included in each group. The results are presented as the mean ± SD. ^∗^*P* < 0.05 and ^∗∗^*P* < 0.01 vs. the corresponding N group.

To evaluate the effect of a high-fat diet on the general characteristics and fertility of the mice, some mice were sacrificed after 8 weeks of feeding, and relevant parameters were examined. As shown in Figure 1(a), male C57BL/6 mice fed the high-fat diet gained significantly more body weight than the mice fed a normal diet (47.25 ± 1.39 vs. 28.14 ± 2.25, *P* < 0.01), which is consistent with the general pictures shown in Figure 1(b). Accompanied by weight gain, there was a significant increase of epididymal adipose in high-fat-diet mice (0.24 ± 0.10 vs. 1.37 ± 0.13, *P* < 0.01, Figure 1(d)), although there was no change in testicular weight (Figure 1(c)). These findings demonstrated that an obese mouse model induced with a high-fat diet was successfully established.

**Figure 2:**
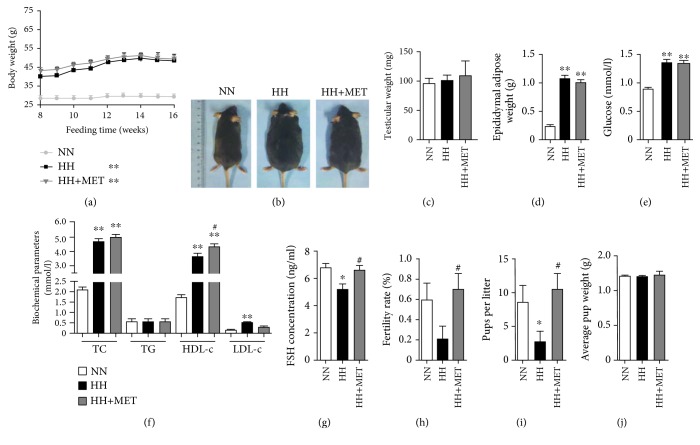
The effects of metformin on serum lipids, glucose, and FSH in high-fat-diet-induced obese male mice. (a) Comparison of the time-dependent changes in body weight among the NN, HH, and HH+MET groups during the 16 weeks of feeding. (b) Representative pictures of mice in each group on the 16th week. Comparison of testicular weights (c) and epididymal adipose tissue (d) of each group of mice on the 16th week. The serum glucose (e), serum lipid parameters (f), and FSH levels (g) were assessed on the 16th week of feeding. *n* = 10 for each group. Comparison of fertility, including the fertility rate (h), number of pups per litter (i), and average pup weight (j) on the 16th week of feeding. For the fertility assay, *n* = 5 males were included in each group. The results are presented as the mean ± SD. ^∗^*P* < 0.05 and ^∗∗^*P* < 0.01 vs. the corresponding NN group; ^#^*P* < 0.05 vs. the corresponding HH group.

Obesity is often accompanied by dyslipidaemia [33], which is defined as an increase of plasma TG and LDL-c or a decrease of plasma HDL-c [33, 34], or by hyperglycaemia [35]. Thus, the serum lipid profile and Glu in the obese mouse model were assessed. The results showed that the serum Glu levels and serum TC, HDL-c, and LDL-c in the high-fat-diet-fed mice increased significantly relative to those in the normal-diet-fed mice (Glu, 6.91 ± 1.38 vs. 12.13 ± 1.82; TC, 1.85 ± 0.13 vs. 3.08 ± 0.39; HDL-c, 1.65 ± 0.14 vs. 2.71 ± 0.38; LDL-c, 0.22 ± 0.04 vs. 0.41 ± 0.11, *P* < 0.01), but there was no difference in serum TG (Figures 1(e) and 1(f)). These alterations are consistent with the results during the 16th week of feeding (Figures 2(e) and 2(f)). These results indicated that glucose and lipid metabolism was highly disrupted in the high-fat-diet-induced obese mouse model.

FSH regulates spermatogenesis and the function of SCs in mammals. To analyze whether the high-fat diet affects FSH, the level of serum FSH was measured. As shown in Figure 1(g), the serum FSH levels were lower in the high-fat-diet-fed mice than in the normal-diet-fed mice (5.39 ± 0.67 vs. 3.58 ± 1.43, *P* < 0.05). These results indicated that FSH was abnormal in the high-fat-diet-induced obese mouse model.

To further assess the effect of the high-fat diet on the fertility of the male mice, the reproductive abilities of the male mice with different diets were assessed via cohabitation with female mice. No difference in the fertility rate or the number of pups per litter was observed between the high-fat-diet-fed mice and the normal-diet-fed mice (Figures 1(h) and 1(i)). The average pup weight of the offspring of the high-fat-diet-fed mice was significantly lower than that of the offspring of the normal-diet-fed mice (1.36 ± 0.12 vs. 0.98 ± 0.09, *P* < 0.01, Figure 1(j)), suggesting that a high-fat diet might affect the offspring's size.

### 3.2. Metformin Ameliorates the Decrease in Male Fertility Caused by a High-Fat Diet Independently of the Regulation of Serum Glucose and Lipids

To investigate the effect of metformin on male fertility, the general characteristics, serum parameters, and fertility of mice were assessed after another 8 weeks of oral administration of metformin. As shown in Figures 2(a) and 2(b), the body weight of the oral metformin group did not decrease compared with that of the group fed the high-fat diet alone. Consistent with the body weight, there were no differences in the testicular and epididymal adipose weights between the high-fat-diet group and the oral metformin group (Figures 2(c) and 2(d)). These results indicated that metformin did not ameliorate the weight gain or epididymal adipose accumulation caused by the high-fat diet during the additional 8 weeks of dosing. During the 16th week, serum Glu, TC, HDL-c, and LDL-c levels were increased significantly in the high-fat-diet-fed mice relative to those in the normal-diet-fed mice (Glu, 8.86 ± 0.63 vs. 13.52 ± 3.11; TC, 2.07 ± 0.33 vs. 4.57 ± 0.77; HDL-c, 1.79 ± 0.32 vs. 3.63 ± 1.05; LDL-c, 0.16 ± 0.03 vs. 0.47 ± 0.10, *P* < 0.01), whereas these parameters did not differ between the high-fat-diet-fed mice and the oral metformin mice except HDL-c (a kind of “positive” cholesterol) [33, 36] (3.63 ± 1.05 vs. 4.30 ± 0.67, *P* < 0.05, Figures 2(e) and 2(f)). These results indicated that metformin did not improve glucose and “adverse” serum lipid [33] metabolism at the present dosage and duration. However, compared with the serum FSH levels of the high-fat-diet-fed mice, those of the oral metformin mice were restored to normal levels (5.21 ± 0.86 vs. 6.60 ± 0.65, *P* < 0.05) (Figure 2(g)). Intriguingly, although low-dosage metformin had no effect on high-fat-diet-induced dyslipidaemia or glucose, the reproductive ability assay showed that the fertility rate and number of pups per litter in the oral metformin group were significantly higher than those in the high-fat-diet group (fertility rate, 0.20 ± 0.42 vs. 0.70 ± 0.48; number of pups per litter, 2.60 ± 3.58 vs. 10.40 ± 4.67, *P* < 0.05) and close to the normal level (Figures 2(h) and 2(i)). These results indicated that the decline in male fertility induced by a high-fat diet could be ameliorated through metformin treatment that independently regulated serum glucose and adverse lipids.

### 3.3. Metformin Improves High-Fat-Diet-Induced Testicular Lipid Deposition and Morphological Abnormalities

**Figure 3:**
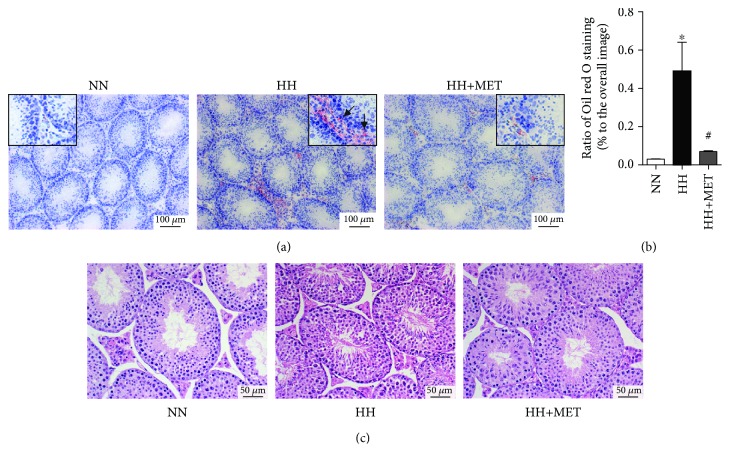
Metformin improves high-fat-diet-induced testicular lipid deposition and morphological abnormalities. (a) Oil red O-stained testicular sections on the 16th week of feeding. The boxed images are enlarged views of each section. The sections of seminiferous tubules are both in spermatogenic stage VIII. Scale bars = 100 *μ*m. *n* = 3 for each group. (b) Ratio of Oil red O staining in seminiferous tubules (% to the overall image). The results are presented as the mean ± SD. ^∗^*P* < 0.05 vs. the corresponding NN group; ^#^*P* < 0.05 vs. the corresponding HH group. (c) H&E-stained testicular sections on the 16th week of feeding. *n* = 3 for each group. Scale bars = 100 *μ*m. All of the above micrographs are representative of 3 independent experiments.

To determine the effect of the high-fat diet on lipid content in the testis, Oil red O staining was performed to visualize lipid deposition in the same cycle of the seminiferous epithelium. The testis sections of the high-fat-diet-fed mice exhibited abundant lipid content, mainly in the interstitial portion, and the lipid ectopic accumulation in seminiferous tubules was also increased. The testicular lipid deposition in seminiferous tubules of the oral metformin mice was clearly lower than that in the high-fat-diet-fed mice (0.49 ± 0.26 vs. 0.07 ± 0.01, *P* < 0.05) (Figures 3(a) and 3(b)). These observations indicated that prior to the improvements in serum lipids and glucose, treatment with metformin alleviated ectopic lipid deposition in the testis.

To further assess the changes in the seminiferous tubules following exposure to a high-fat diet and oral metformin in mice, testicular morphology was analyzed by H&E staining. As shown in Figure 3(c), the seminiferous epithelia in the high-fat-diet group were severely disorganized, and cell adhesion between SCs and spermatogenic cells was disrupted and loosely arranged. After treatment with oral metformin, the spermatogenic epithelial morphology was improved, and the adhesion between cells was similar to that between cells in the normal-diet group. These findings demonstrated that metformin improved the high-fat-diet-induced disruption in the general structure of the seminiferous tubules.

### 3.4. Metformin Repairs the BTB Integrity Disruption Induced by a High-Fat Diet

**Figure 4:**
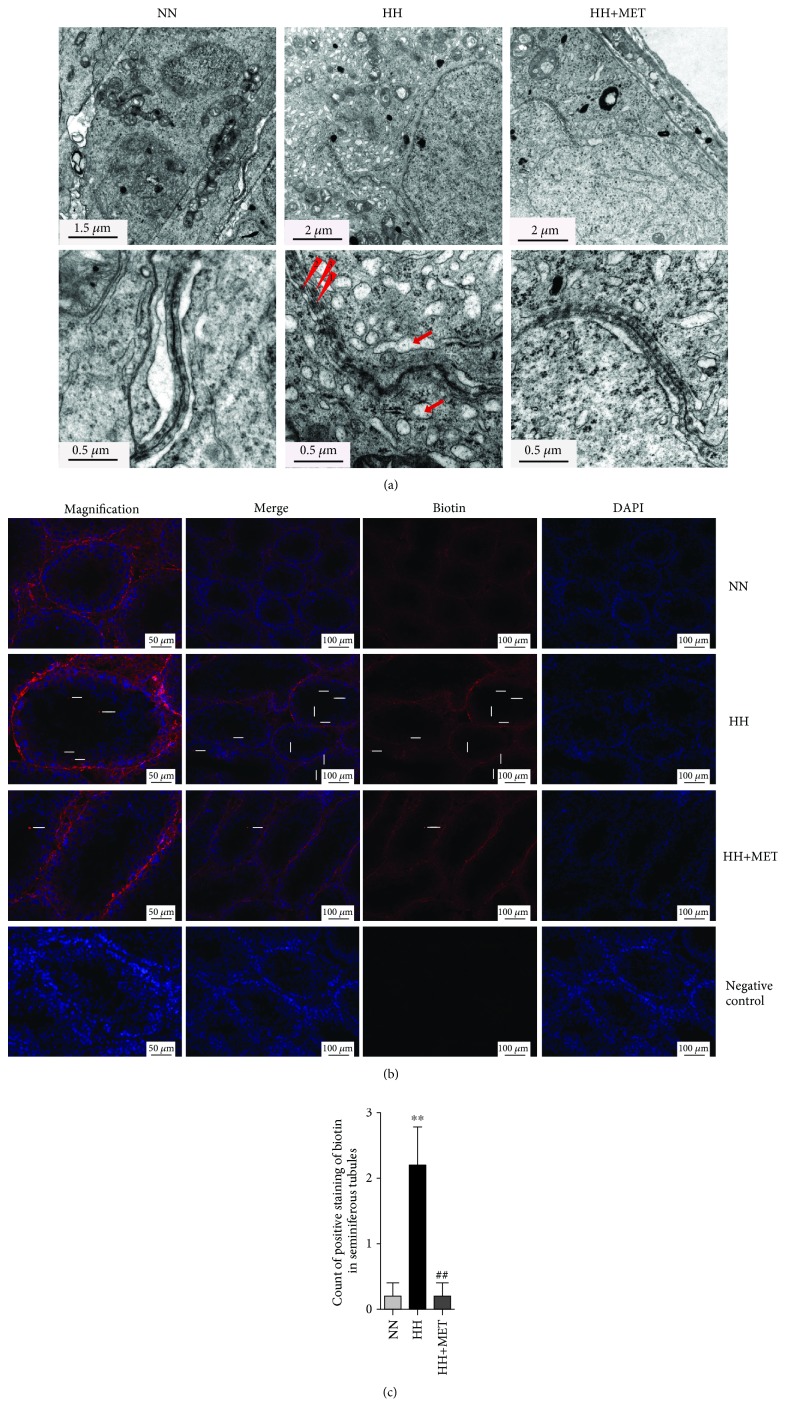
Metformin repairs the disruption of BTB integrity induced by a high-fat diet. (a) Transmission electron micrographs of the seminiferous epithelia of mice on the 16th week of feeding; the lower images are magnified views of each of the paired sections above. Red arrowhead 1 shows the cracked BTB between two adjacent Sertoli cells constituted by cellular tight junctions. Red arrowheads 2 and 3 indicate the increase in protein along both sides of the BTB. The red arrows represent expanded endoplasmic reticula. The corresponding scale bar is marked in the lower left corner of each image. *n* = 3 for each group. (b) On the 16th week of feeding, testes were injected with 50 *μ*l of EZ-Link Sulfo-NHS-LC-Biotin (red), and cell nuclei were stained with DAPI (blue). In the sections of testes from NN mice, the red fluorescence of the biotin tracer was observed only in the interstitial spaces and basal compartment. In the HH + MET group, a small amount of red fluorescence was seen in the lumina of the seminiferous tubules (white arrow), whereas in the HH group mice, abundant red fluorescence was evident in the lumina of the seminiferous tubules (white arrows) beside the interstitial space and basal compartment. Scale bars = 50 *μ*m for the magnified images, and scale bars = 100 *μ*m for the other images. *n* = 3 for each group. All of the above micrographs are representative of 3 independent experiments. (c) Count of positive staining of biotin in seminiferous tubules. The results are presented as the mean ± SD. ^∗∗^*P* < 0.01 vs. the corresponding NN group; ^##^*P* < 0.01 vs. the corresponding HH group.

The integrated BTB structure is one of the tightest blood-tissue barriers in the body, and it is crucial for maintaining cell adhesion between the opposing SCs and adjacent Sertoli-germ cells. To characterize the BTB morphology at the ultrastructural level, a TEM analysis of the testis from each group of mice was performed. As depicted in Figure 4(a), the BTBs in the normal-diet-fed mice and oral metformin mice were lined with endoplasmic reticulum (ER) cisternae and clearly delimited, continuous, and tight. In contrast, the cell junctions adjoining SCs appeared discontinuous and dehisced in the seminiferous tubules from the high-fat-diet-fed mice. These data showed that metformin repaired the injured integrity of the BTB, which was compromised severely by the high-fat diet. Furthermore, ER expansion and increased proteins alongside the BTB were found in the high-fat-diet-fed mice. The black particles beside the TJs, appearing as a net-like meshwork, represent polymers of interacting junctional transmembrane proteins [37]. These results indicated the possible existence of ER stress and the abnormal expression of BTB-related proteins.

Next, to further explore the effect of lipotoxicity on BTB integrity and the effect of metformin in the process, we analyzed BTB integrity with a biotin tracer (Figures 4(b) and 4(c)). In normal testes, junctions between the Sertoli-Sertoli and Sertoli-spermatid interfaces formed tight barriers that prevented large molecules from passing through. However, the BTB was opened after 16 weeks on the high-fat diet. Red fluorescent dye that was injected into the interstitium of the testes diffused into the BTB and appeared abundantly in the adluminal compartment. Additionally, only a small amount of red fluorescence appeared in the adluminal compartment of the HH+MET mice (2.20 ± 1.30 vs. 0.20 ± 0.45, *P* < 0.01) (Figure 4(b), HH vs. HH+MET). These results indicated that the integrity of the BTB in the testes of the high-fat-diet-fed mice was indeed disrupted and metformin essentially repaired this disruption.

### 3.5. Metformin Recovers the Disruption in Junction Protein Expression Induced by a High-Fat Diet

**Figure 5:**
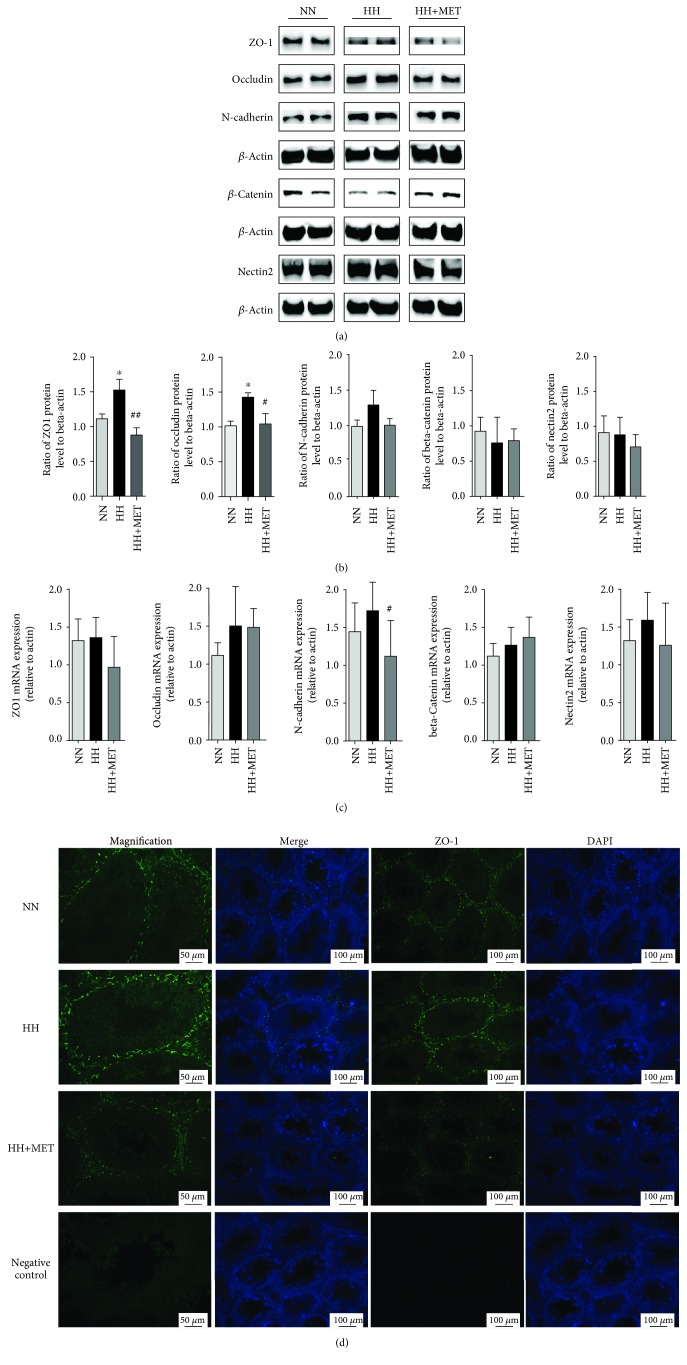
Metformin recovers the disordered junction protein expression induced by a high-fat diet. (a) The ZO-1, occludin, N-cadherin, *β*-catenin, and nectin2 bands in the immunoblotting analysis of the mouse testes. Testis lysates (120 *μ*g/well) were loaded onto gels, electrotransferred onto membranes, and reacted with primary and secondary antibodies; beta-actin was used as the loading control. All gels were run under the same experimental conditions. The lower panel (b) is a densitometric histogram of the protein bands, and the results are expressed as the ratio of the corresponding protein to beta-actin. (c) The mRNA expression of ZO-1, occludin, N-cadherin, *β*-catenin, and nectin2 in mouse testes; gene expression was normalized to beta-actin. ^∗^*P* < 0.05 vs. the corresponding N group; ^#^*P* < 0.05 and ^##^*P* < 0.01 vs. the corresponding HH group. *n* = 3 for each group. (d) Immunofluorescence micrographs using cross-sections of testes. Green fluorescence represents ZO-1, and blue (DAPI dye) represents the nuclei. Scale bars = 50 *μ*m for the magnified images, and scale bars = 100 *μ*m for the other images. *n* = 3 for each group. All of the above panels are representative of 3 independent experiments.

The integrity of the BTB is based on various junction proteins that form coexisting TJs and ectoplasmic specializations. To further verify the damage to the BTB induced by the high-fat diet and the protective effect of metformin, the mRNA and protein levels of BTB-related molecules were detected on the 16th week. Compared with the levels in the testis of the normal-diet group, although there was no significant change in the mRNA levels, TJ integral membrane proteins including ZO-1 and occludin were upregulated significantly in the testis of the high-fat-diet group (ZO-1, 1.11 ± 0.14 vs. 1.53 ± 0.31, *P* < 0.05; occludin, 1.02 ± 0.13 vs. 1.43 ± 0.13, *P* < 0.05). Metformin improved the abnormal protein expression of ZO-1 and occludin (ZO-1, 1.53 ± 0.31 vs. 0.88 ± 0.20, *P* < 0.01; occludin, 1.43 ± 0.13 vs. 1.04 ± 0.30, *P* < 0.05) (Figures 5(a)–5(c)). These results indicated that the modification of ZO-1 and occludin protein synthesis might be related to the protective effect of metformin on BTB damage induced by a high-fat diet.

In addition to the abnormalities in junction protein expression, the localization of the junction protein on the BTB affects the function of the BTB. To further verify the expression and localization of junction proteins on the BTB in testicular SCs, immunofluorescence of ZO-1 was performed. The high-fat diet increased ZO-1 accumulation on the BTB; metformin recovered the increase in ZO-1 on the BTB (Figure 5(d)), which was consistent with testicular ultrastructure and immunoblotting analyses of the testis. However, the localization of ZO-1 on the BTB was unaltered.

### 3.6. Metformin Suppresses the Oxidative Stress Level in the Testis via Inhibition of NF-*κ*B/p65 Activity in SC Nuclei

**Figure 6:**
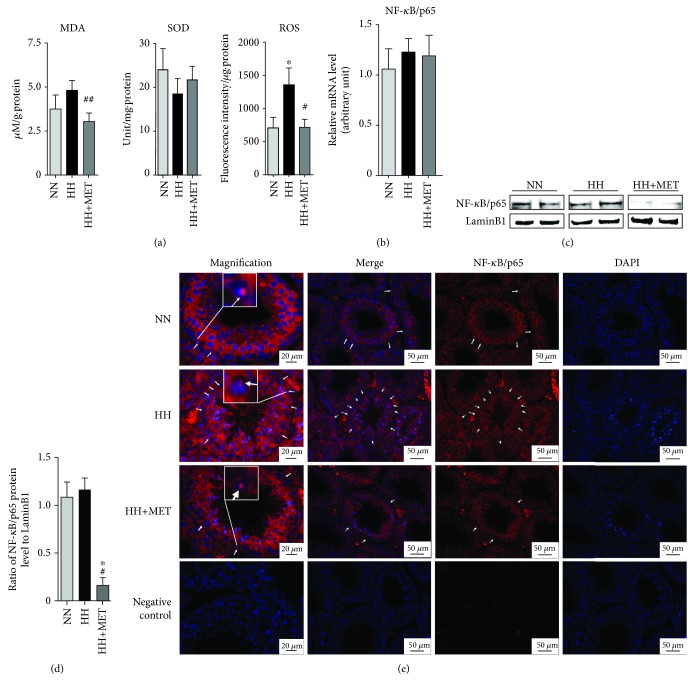
Metformin suppresses the oxidative stress level in the testis via inhibition of NF-*κ*B/p65 in SC nuclei. (a) The levels of MDA, SOD, and ROS in each group. All contents and enzyme activities were normalized to the total protein level, which was measured by means of the BCA method. ^∗^*P* < 0.05 vs. the corresponding NN group; ^#^*P* < 0.05 and ^##^*P* < 0.01 vs. the corresponding HH group. *n* = 3 for each group. (b) The mRNA expression of NF-*κ*B/p65 in mouse testes; gene expression was normalized to beta-actin. *n* = 3 for each group. (c) The NF-*κ*B/p65 bands in the immunoblotting analysis of the mouse testes. Testicular nucleoprotein lysates (60 *μ*g/well) were loaded onto gels, electrotransferred onto membranes and reacted with primary and secondary antibodies; LaminB1 was used as the loading control. *n* = 3 for each group. (d) A densitometric histogram of the NF-*κ*B/p65 protein bands; the results are expressed as the ratio of the corresponding protein to LaminB1. ^∗^*P* < 0.05 vs. the corresponding NN group; ^##^*P* < 0.01 vs. the corresponding HH group. (e) Immunofluorescence micrographs using cross-sections of deparaffinized testes. *n* = 3 for each group. Red fluorescence represents NF-*κ*B/p65, and blue fluorescence (DAPI dye) represents the nuclei. The white arrows represent the positively stained nuclei of Sertoli cells. A nucleus with the colocalization of DAPI and nuclear NF-*κ*B was magnified in 20 *μ*m images, which was framed in a white box. Scale bars = 20 *μ*m for the magnified images, and scale bars = 50 *μ*m for the other images. All of the above bands or micrographs are representative of 3 independent experiments.

Obesity can increase oxidative stress in the whole body [25], and an increase in testicular oxidative stress is a common feature in much of what underlies male infertility [26]. Therefore, to evaluate whether metformin improves male mouse fertility by alleviating oxidative stress, testicular oxidative stress-related indexes were assessed in each group on the 16th week, and the indexes included MDA, an index of lipid peroxidation and one of the commonly used biomarkers of oxidative stress [38]; SOD, a type of scavenger of oxygen radicals [39] and a type of critical antioxidant enzyme that protects cells from oxidative stress [40]; and ROS, a kind of oxidative stress inducer species. As shown in Figure 6(a), although there was no difference in SOD activity, the fluorescence intensity of ROS was significantly higher in the testes of the high-fat-diet-fed mice than in those of the normal-diet-fed mice (705.3 ± 161.7 vs. 1357 ± 254.8, *P* < 0.05). In the testes of the oral metformin mice, the fluorescence intensity of ROS (1357 ± 254.8 vs. 716.1 ± 119.8, *P* < 0.05) and concentration of MDA (4.81 ± 0.56 vs. 3.04 ± 0.49, *P* < 0.01) significantly decreased to the normal level. These results indicated that metformin alleviated high-fat-diet-induced excess oxidative stress in the testes.

Oxidative stress leads to inflammation, a process that is most strongly influenced by the level of oxidative stress and mediated primarily by NF-*κ*B, which is a proinflammatory factor. To further verify whether NF-*κ*B was involved in the process, the protein in nuclei and mRNA expression levels of NF-*κ*B/p65 in the testis were assessed. Metformin treatment resulted in a significant decrease of the expression level of NF-*κ*B/p65 in the whole testis (1.09 ± 0.16 vs. 0.16 ± 0.08 and 1.17 ± 0.12 vs. 0.16 ± 0.08, *P* < 0.05) (Figures 6(c) and 6(d)). However, high-fat diet and metformin did not affect NF-*κ*B/p65 mRNA expression in the whole testicular tissue (Figure 6(b)). NF-*κ*B is normally located in the cytoplasm, and activated NF-*κ*B localizes to the nucleus. To further represent the level of NF-*κ*B/p65 in SC nuclei, NF-*κ*B expression in SC nuclei was detected through an immunofluorescence method using cross-sections of deparaffinized testes. As shown in Figure 6(e), red fluorescence in the SC nuclei represents activated NF-*κ*B. The greatest abundance of activated NF-*κ*B in the SC nuclei existed in the high-fat-diet-fed mice, and the abundance in the oral metformin mice was similar to that in the normal-diet-fed mice. These results indicated that metformin reduced the expression of NF-*κ*B/p65 in the SC nuclei, which might be related to the reduction in excess oxidative stress in the testes induced by the high-fat diet.

## 4. Discussion

Obesity, which is associated with male infertility, has increased dramatically worldwide. Metformin, a commonly used hypoglycaemic agent, has been shown to play roles in nondiabetic diseases through antioxidant effects. However, whether metformin can improve the damage to male fertility caused by obesity remains unclear. In this study, by constructing a high-fat-diet-induced obese male mouse model and administering metformin in water, we demonstrated that metformin reduced the high level of oxidative stress in the testis of high-fat-diet-fed obese mice before improvements in serum glucose and adverse lipids. In addition to the improvements in oxidative stress in the testis, the disrupted expression of junction proteins was ameliorated, the injured structure and integrity of the BTB were amended, and fertility was improved by metformin. These results suggested that metformin improved the reproductive function of obese male mice via an antioxidant effect independently of its regulation of serum glucose and lipids.

Metformin, a widely used medicine for the treatment of type 2 diabetes mellitus, has extensive protective effects on many organs and cells, such as the vessels, heart, brain, and ovaries [41–44]. Moreover, metformin can improve the reproductive function of obese females among patients with PCOS in clinical applications [45]. However, the role of metformin in the male gonads is controversial. An *in vitro* study has demonstrated that metformin played a beneficial role in energy metabolism in SCs [46]. Furthermore, metformin stimulates alanine production in SCs, inducing antioxidant activity and maintaining the NADH/NAD+ equilibrium. However, other studies have reported that metformin might disrupt normal testicular physiological processes, leading to spermatogenic failure [47]. These are indeed crucial results demonstrating the detrimental effects of metformin, which are apparently contradictory to the results presented here. Apart from the experimental methods used, the results of the previous study were obtained from healthy animals with apparently normal serum glucose and lipid metabolism. However, in our study, the effect of metformin was analyzed under hyperlipidaemic and hyperglycaemic conditions during adulthood in mice, which may explain the conflicting results when compared with other research.

The negative impact of obesity on the male genital system is gradually being recognized. Obesity-induced decreases in male fertility have been demonstrated in animal models and clinical investigations, and abnormalities in pituitary-gonadal axis hormones have been demonstrated in morbidly obese men [48, 49]. In our study, the high-fat diet resulted in decreased fertility in obese male mice, and further experiments demonstrated that the decreased fertility was accompanied by abnormalities in lipid metabolism and sex hormones. Apart from dyslipidaemia in obese male mice, ectopic lipid accumulation was abundant in the testicular interstitium, and some of the lipids penetrated the basement membrane and deposited ectopically in the basement compartment of the seminiferous tubules; the structure and integrity of the BTB were destroyed. This result demonstrates that lipotoxicity indeed injured the BTB, and the testis/seminiferous tubules were in a lipotoxic microenvironment. Of note, testicular lipid deposition did not increase testicular weight, which might be due to organ specificity of the testis or high-fat diet for a short period of time. In male peripheral gonads, lipid accumulation mainly deposited in the epididymis and surrounded testis, and a longer-term high-fat diet will be given in further study. Metformin played a substantial role in regulating lipid metabolism, and the 8-week treatment with metformin markedly reduced the ectopic lipid deposition in the testis; however, serum glucose and adverse lipids were not influenced significantly, which might be due to the lower dosage of metformin. Relative to the dose of metformin (200 mg/kg/d) used in this experiment, the dosage for humans suggested in the control of diabetes is 2000 mg/d (body weight = 70 kg); correspondingly, the dosage of metformin that might reduce serum glucose in male mice should be 260 mg/kg/d (9.1 × 2000 mg/d/70 kg). To further verify this speculation, a higher dosage of metformin will be administered in further study. However, the data also suggest that metformin prioritizes the adjustment of lipid metabolism in the testicular tissue before regulating serum glucose and lipids. In addition to abnormalities in lipid metabolism, changes in gonadotropin levels were also analyzed; FSH, which decreased significantly in the high-fat-diet-fed mice, plays an important role in spermatogenesis via the FSH receptor (FSHR) [50]. With the decline in FSH, fertility decreased and the BTB was impaired in the high-fat-diet-fed mice. Fertility and the integrity of BTB were recovered with the increase in FSH in the mice-administered metformin. These results indicate that a high-fat diet has a detrimental effect on pituitary gonadotropin FSH, which might also occur via lipotoxicity in the pituitary. Metformin, in addition to directly affecting the peripheral target gonad, can improve reproductive capacity by regulating the centre of the gonadal axis.

Previous studies on the mechanisms of male fertility have mainly focused on sperm parameters, Leydig cells, or reproductive hormones [9, 51–53]. The function of the SCs was overlooked. SCs provide nutrition and BTB support in spermatogenesis. Disrupting BTB integrity harms the reproductive system and results in reproductive dysfunction [19, 54]. As mentioned above, in the high-fat-diet-fed mice, abundant lipids were deposited ectopically in the testicular interstitium, and some lipids were deposited ectopically in the seminiferous tubules, indicating lipotoxicity in the testis or seminiferous tubules. In the high-fat-diet-fed mice, the expression of TJ-related proteins (ZO-1 and occludin) increased significantly, which were basically consistent with the previous studies [55]. Morgan et al. [55] found that the upregulation of ZO-1 and occludin was a compensation for BTB damage. Such changes might be an adaptive change because SCs increasingly synthesize tight-junction proteins and attempt to repair the dehiscent BTB. Disordered junction protein expression leads to injury to the structure and integrity of the BTB. TJ-associated proteins may be the targets of lipotoxicity, and this is consistent with previous studies of the effects of other exogenous stimulators on SC BTB, such as perfluorooctanesulfonate, cadmium, amodiaquine, and bisphenol A [19–22, 24]. In oral metformin mice, lipid accumulation in the testicular interstitium and seminiferous tubules decreased dramatically. In addition, with a concomitant decrease in ectopic lipid deposition, the disordered junction protein expression, damaged structure, and integrity of the BTB were ameliorated, and the decrease in fertility was resolved. These results indicate that metformin can directly regulate testicular lipid metabolism and hence reverse the injury to the BTB, improving decreased fertility.

Metformin has antioxidant properties. Previous studies have demonstrated that metformin can reduce ROS [56] and that, partly by reducing oxidative stress inducer species, metformin can confer health and lifespan benefits to laboratory mice and *C. elegans* [57, 58]. The high-fat-diet-fed mice exhibited decreased reproductive function together with increased ROS. Previous studies have demonstrated that hydrogen peroxide, the most abundant ROS in pathophysiology, can reduce the expression of occludin, a marker TJ protein and a principal target of redox processes, in cultured MDCK-II cells and that O_2_^−^ can decrease occludin expression in cultured brain endothelial cells [59]. ROS also modulates the patterns of gene expression through functional alterations to transcription factors, such as NF-*κ*B [60]. NF-*κ*B activation is required in TNF-*α*-downregulated ZO-1 expression in the intestinal TJ barrier [61], and NF-*κ*B inhibitors (caffeic acid phenethyl ester) can recover the decreased expression of occludin and ZO-1 induced by methotrexate in the intestinal barrier [62]. In other words, NF-*κ*B is involved in the regulation of the expression of TJ-related proteins in other blood-tissue barriers and may regulate the expression of BTB-related proteins. Likewise, our data demonstrate that the level of ROS is significantly higher in high-fat-diet-fed mice than in normal-diet-fed mice and that NF-*κ*B is activated abundantly in high-fat-diet-fed mice compared with normal-diet-fed mice. Thus, the activated ROS-NF-*κ*B signalling pathway might be a contributor to high-fat-diet-induced BTB damage. Inhibition of NF-*κ*B signalling represents a viable strategy for disease therapy [63]. The levels of ROS and the abundance of activated NF-*κ*B/p65 in SC nuclei were significantly lower in the mice administered metformin than in the high-fat-diet-fed mice. This indicates that treatment with metformin reduces ROS levels, inhibits the activity of NF-*κ*B in SC nuclei, likely ameliorates lipotoxicity-induced disruption of BTB-associated proteins, and further repairs the structure and integrity of the BTB, upregulating the decrease in the fertility of obese male mice. All of these observations point towards crosstalk between an ROS-NF-*κ*B mediator and the intervening role of metformin in determining BTB function in a testicular fat ectopic accumulation microenvironment. The studies performed by Bonnefont-Rousselot et al. [64] and Esteghamati et al. [65] demonstrated that metformin has a remarkable effect on oxidative stress. Our study further demonstrated that metformin inhibits the activity of NF-*κ*B in the SC nuclei of obese male mice and hence exerts a beneficial effect on the BTB.

## 5. Conclusion

**Figure 7:**
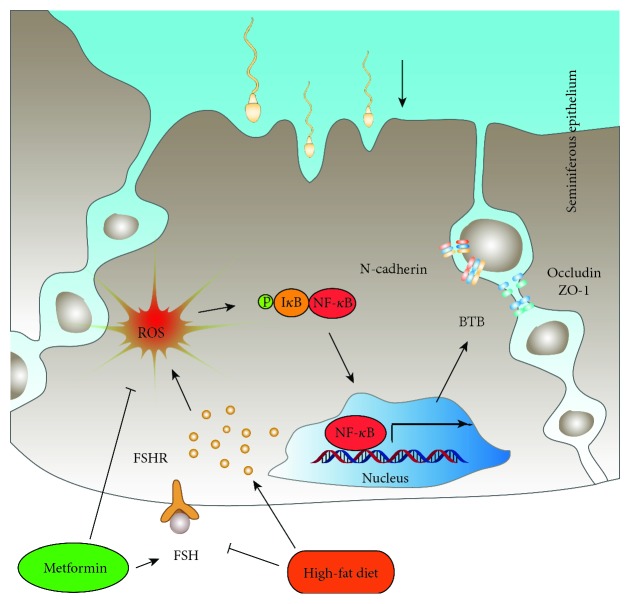
Model accounting for the mechanism of the metformin-mediated improvement of fertility in obese male mice. In mice on the high-fat diet, the ectopic deposition of adipose in the testis increases the level of oxidative stress, especially the level of ROS, activating NF-*κ*B in the cytoplasm, resulting in an increase in NF-*κ*B in the nuclei of SCs. The latter results in disordered junction protein expression, which leads to the destruction of BTB integrity and then the decline in spermatogenesis and male fertility. Metformin reduces lipid deposition in the testis, relieves oxidative stress in the testis, inhibits excessive activation of NF-*κ*B in SC nuclei, reverses the destruction of the BTB, and hence improves the reproductive function of male mice. In other words, metformin ameliorates the injury to the reproductive function of obese male mice via the ROS-NF-*κ*B pathway. In addition, FSH is decreased in high-fat-diet-fed male mice, and metformin increases the level of FSH in obese male mice, which might play a role in the process of the metformin-mediated improvement of fertility in obese male mice.

In summary, the results of this study confirm that a high-fat diet causes abnormalities in glucose and lipid metabolism and serum FSH, increases oxidative stress, and disrupts BTB integrity in the testis, harming the reproductive system. Treatment with metformin decreases ectopic lipid accumulation in the testis, reduces the levels of oxidative stress, ameliorates the high-fat-diet-induced injury to the BTB, and improves fertility in obese male mice. The ROS-NF-*κ*B pathway may be a mechanism of metformin in this protective process (a diagram is shown in Figure 7). However, the mechanism of metformin is highly complex, and further studies are needed, including an examination of the mechanism underlying the effects of metformin on pituitary gonadotropin FSH and NF-*κ*B signalling. These results are important evidence of the beneficial effects of metformin on infertility in obese males and provide an effective treatment option for male infertility in obese men. Clinical trials will be analyzed to further confirm the beneficial effects of metformin on the fertility of obese men.

## Figures and Tables

**Table 1:** Antibodies.

Antibody	Species	Dilution	Corporation	Catalogue number
ZO-1	Rabbit	1 : 50	Thermo Fisher Scientific	40-2200
Occludin	Rabbit	1 : 62.5	Thermo Fisher Scientific	71-1500
N-cadherin	Rabbit	1 : 500	Proteintech Group Inc.	13769-1-AP
beta-Catenin	Rabbit	1 : 1000	Proteintech Group Inc.	51067-2-AP
Nectin2	Rabbit	1 : 10000	Abcam	Ab135246
NF-*κ*B/p65	Rabbit	1 : 2500	Abcam	ab32536
beta-Actin	Rabbit	1 : 7500	Proteintech Group Inc.	60008-1
LaminB1	Mouse	1 : 7500	Proteintech Group Inc.	66095-1

**Table 2:** Primers for real-time PCR detection.

Gene	Species	Forward primer	Reverse primer
ZO-1	Mouse	GCCGCTAAGAGCACAGCAA	TCCCCACTCTGAAAATGAGGA
Occludin	Mouse	TTGAAAGTCCACCTCCTTACAGA	CCGGATAAAAAGAGTACGCTGG
N-cadherin	Mouse	AGCGCAGTCTTACCGAAGG	TCGCTGCTTTCATACTGAACTTT
beta-Catenin	Mouse	CCCAGTCCTTCACGCAAGAG	CATCTAGCGTCTCAGGGAACA
Nectin2	Mouse	GCATCATTGGAGGTATTATCGCT	GAGGGAGGTCCTTCCAGTTC
NF-*κ*B/p65	Mouse	CGGGATGGCTACTATGAGGC	CGTGAAAGGGGTTATTGTTGGT
beta-Actin	Mouse	GGCTGTATTCCCCTCCATCG	CCAGTTGGTAACAATGCCATGT

## Data Availability

The data used to support the findings of this study are included within the article.

## Abbreviations

FSH: Follicle-stimulating hormone
BTB: Blood-testis barrier
MDA: Malondialdehyde
SOD: Superoxide dismutase
ROS: Reactive oxygen species
NF-*κ*B: Nuclear factor *κ*B
SCs: Sertoli cells
BMI: Body mass index
TJs: Tight junctions
Basal ESs: Basal ectoplasmic specializations
PCOS: Polycystic ovarian syndrome
MDF: Modified Davidson's fluid
TC: Total cholesterol
TG: Triglycerides
HDL-c: High-density lipoprotein-cholesterol
LDL-c: Low-density lipoprotein-cholesterol
Glu: Glucose
H&E: Haematoxylin and eosin
TEM: Testicular transmission electron microscopy
PFA: Paraformaldehyde
PBST: Phosphate-buffered saline with 0.1% Tween20
PBS: Phosphate buffer solution
BSA: Bovine serum albumin
DAPI: 4′-6-Diamidino-2-phenylindole
HRP: Horseradish peroxidase
PCR: Polymerase chain reaction
ER: Endoplasmic reticulum
FSHR: FSH receptor.

## Supplementary Materials

Supplementary Materials Figure S1: the flowchart of the animal experiments. One week after feeding to adapt to the housing conditions, the male mice were randomly divided into two groups: the normal-diet group (N, *n* = 20) was fed a standard diet, and the high-fat-diet group (H, *n* = 30) was fed a high-fat diet. Ten mice from each group were sacrificed at the end of the 8th week of feeding. The remaining mice in the N group were maintained on their standard diet (NN, *n* = 10), whereas the mice fed a high-fat diet (*n* = 20) were further subdivided into two subgroups. The first subgroup (HH, *n* = 10) was maintained on the high-fat diet, and the second subgroup (HH + MET, *n* = 10) was maintained on the high-fat diet with ~200 mg/kg body weight/day metformin given in the drinking water.
