# Supplementary figures and images for: Metformin Improves Fertility in Obese Males by Alleviating Oxidative Stress-Induced Blood-Testis Barrier Damage
Source: Oxid Med Cell Longev. 2019 Sep 10;2019:9151067. doi: 10.1155/2019/9151067 (PMC6754953; doi:10.1155/2019/9151067)

**Supplemental data**

**Figure S1: The flowchart of the animal experiments**


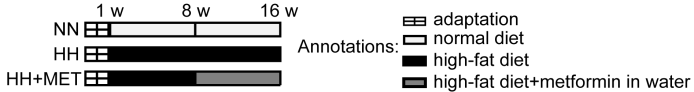

Supplement: Supplementary Materials — Figure S1: the flowchart of the animal experiments. One week after feeding to adapt to the housing conditions, the male mice were randomly divided into two groups: the normal-diet group (N, n = 20) was fed a standard diet, and the high-fat-diet group (H, n = 30) was fed a high-fat diet. Ten mice from each group were sacrificed at the end of the 8th week of feeding. The remaining mice in the N group were maintained on their standard diet (NN, n = 10), whereas the mice fed a high-fat diet (n = 20) were further subdivided into two subgroups. The first subgroup (HH, n = 10) was maintained on the high-fat diet, and the second subgroup (HH + MET, n = 10) was maintained on the high-fat diet with ~200 mg/kg body weight/day metformin given in the drinking water. [file 9151067.f1.zip › mat.9151067.v3.docx]
